# Comprehensive Genetic Analysis of Associations between Obesity-Related Parameters and Physical Activity: A Scoping Review

**DOI:** 10.3390/genes15091137

**Published:** 2024-08-28

**Authors:** Agata Leońska-Duniec

**Affiliations:** Faculty of Physical Education, Gdansk University of Physical Education and Sport, 80-336 Gdansk, Poland; agata.leonska-duniec@awf.gda.pl

**Keywords:** total genetic score, Biofilter software, obesity, gene–physical-activity interaction

## Abstract

Genetic epidemiological studies have shown that numerous genetic variants cumulatively increase obesity risk. Although genetically predisposed individuals are more prone to developing obesity, it has been shown that physical activity can modify the genetic predisposition to obesity. Therefore, genetic data obtained from earlier studies, including 30 polymorphisms located in 18 genes, were analyzed using novel methods such as the total genetic score and Biofilter 2.4 software to combine genotypic and phenotypic information for nine obesity-related traits measured before and after the realization of the 12-week training program. The results revealed six genes whose genotypes were most important for post-training changes—*LEP*, *LEPR*, *ADIPOQ*, *ADRA2A*, *ADRB3*, and *DRD2*. Five noteworthy pairwise interactions, *LEP* × *LEPR*, *ADRB2* × *ADRB3*, *ADRA2A* × *ADRB3*, *ADRA2A* × *ADRB2*, *ADRA2A* × *DRD2*, and three specific interactions demonstrating significant associations with key parameters crucial for health, total cholesterol (TC), high-density lipoprotein (HDL), and fat-free mass (FFM), were also identified. The molecular basis of training adaptation described in this study would have an enormous impact on the individualization of training programs, which, designed according to a given person’s genetic profile, will be effective and safe intervention strategies for preventing obesity and improving health.

## 1. Introduction

Obesity is a chronic, multifactorial disease defined as the accumulation of body fat to the extent that it negatively affects health. Obesity has a well-proven genetic basis but requires behavioral, developmental, and/or environmental influences to develop [1,2,3]. In recent years, the number of people with overweight and obesity has dramatically increased worldwide, representing a significant public health concern. Given the excess mortality, morbidity, and economic toll, obesity is an illness that warrants increased attention from medical, scientific, and community organizations. Obesity’s status and acceptance as a chronic disease are critical in determining its treatment and the development of comprehensive interventions [3]. It has been confirmed that systematic physical activity during a diet-induced weight loss program has profound additional metabolic advantages in people with diabetes and obesity [4,5]. However, exercise recommendations do not account for individual genetic variability, increasing risk of these diseases [6].

Numerous studies have shown that systematic exercise and habitual physical activity have numerous benefits for human health and life expectancy. Physical activity is crucial for reducing the risk of excessive body mass gain, improving the efficiency of fat loss programs, and especially preventing weight regain [7,8]. The physiological and biochemical reactions occurring in the human body after training are well-described. Frequent exercise leads to various metabolic and physical changes, such as alterations in skeletal muscle characteristics, nutrient storage, metabolic enzyme levels, contractile protein quantity, and connective tissue stiffness. The complex process of exercise-induced adaptation is determined by the volume, intensity, and frequency of physical activity [9,10]. In addition, Bouchard (1983) demonstrated significant individual differences in the response to highly standardized and well-controlled exercise programs and that there was a substantial familial aggregation component to the heterogeneity noted above, confirmed by several other authors [11,12,13]. However, few studies have focused on the role of specific genes in accounting for the highly prevalent effects of gene-exercise interaction. These authors indicated that training-induced changes in several physical performance and health-related fitness phenotypes may be more effective in individuals with some genotypes than others, confirming that some gene variants influence individual differences in response to regular exercise [9,11,14]. However, identifying the genetic markers associated with obesity and explaining the complex mechanisms through which they exert their effects pose challenges. In the future, a better understanding of the molecular basis of training adaptation could significantly affect the customization of training programs to make them more effective and safer. It could also improve recovery, trauma care, medical treatment, diet, or supplementation. The specificity of gene–physical-activity interactions is crucial for sports scientists and offers promising pathways for identifying targets to address obesity. This knowledge holds significant potential for informing both athletic performance and therapeutic interventions [9,15].

There have been several studies over the past 30 years on genetic obesity, which have shown that genetic mutations, polymorphisms, and changes in gene expression all play a role in predisposing individuals to obesity [16]. To date, more than 600 genes and chromosomal regions have been linked to body mass and composition regulation. The genetic risk of common obesity is associated with the accumulation of various *loci*, each contributing a small part of the total risk of obesity [17]. Beyond the large number of possible genetic markers, an additional problem lies in determining their influence on lifestyle-induced changes in obesity-related parameters, separately and together. In earlier studies, the associations of the numerous polymorphisms with training-induced changes in body mass, composition, and biochemical parameters in Caucasian women have been analyzed. However, each of those studies involved a single or a small number of single nucleotide polymorphisms (SNPs), which did not allow comparison of the polymorphisms and led to comprehensive conclusions about their impact on the characteristics and range of the body’s adaptive response to training. Therefore, simultaneously analyzing numerous SNPs is more advantageous than other methods and may offer additional insights for comprehending complex gene–physical-activity interactions. Consequently, in this study, genetic data obtained from earlier studies conducted using 30 polymorphic sites located in 18 genes were used as components of a polygenic profile to find all ‘preferable’ and ‘unpreferable’ genotypes for training-induced body changes, which will provide additional information about people undertaking physical training. In addition, Biofilter software was used to construct a novel picture of the relationships among the genetic architecture and proteins, such as interaction pairs, pathways, and complex phenotypic outcomes, as described in previous biological experiments.

## 2. Materials and Methods

### 2.1. Overview

The results obtained from my studies regarding gene–physical-activity interactions were collected, and the functional significance of the genotypes described in the 30 common polymorphic sites connected with obesity risk was determined. The genotypes were assessed regarding their impact on training-induced changes in body mass, body composition, glucose level, and lipid profile in Caucasian females. The functional significance of the individual genotypes was determined based on the various consequences of their presence for achieving the desired health-promoting changes induced by the 12-week training program. Next, the relationships between the genetic variants and metabolic health parameters were studied, providing insights into potential factors influencing individual responses to training interventions.

### 2.2. Participants

This study is based on the previously published data, which examined training-induced changes in obesity-related parameters in participants. The participants were the same as those in the studies listed in Table 1 in the Section 3.

The study group consisted of 165–201 healthy females of Caucasian origin (age: 21 ± 1 year). The following inclusion criteria were used: had a low level of physical activity self-reported with the use of the Global Physical Activity Questionnaire; had no metabolic, neuromuscular, or musculoskeletal disorders; were not using supplements or medications; and were nonsmokers. All participants were expected to maintain a balanced diet based on their dietary plan. They took part in the 12-week (36 training units) low- and high-impact aerobics of increasing intensity preceded by a week-long familiarization stage (3 training units). Before the training, participants of the experiment had their maximum heart rate (HRmax) evaluated, on the basis of a continuous graded test on an electronic cycle ergometer (Oxycon Pro, Erich JAEGER GmbH, Hoechberg, Germany). Exercise intensity was designated using HR monitors to control each individual heart rate. The volunteers were instructed to hold an HR or relative value of HRmax within appointed ranges. Each training unit consisted of a warm-up (10 min), aerobic exercise (43 min), and breathing–relaxing exercise with stretching (7 min). The main part was a combination of two styles including high impact (running, jumping, and hopping, with a variety of flight phases) and low impact (movements with at least 1 foot on the floor at all times). The 12-week program was divided as follows:

- 3 weeks (9 training units), 60 min each, at 50–60% of HRmax, tempo 135–140 BPM;
- 3 weeks (9 training units), 60 min each, at 60–70% of HRmax, tempo 140–152 BPM;
- 3 weeks (9 training units), 60 min each, at 65–75% of HRmax, tempo 145–158 BPM;
- 3 weeks (9 training units), 60 min each, at 65–80% of HRmax, tempo 145–160 BPM.

The training and dietary program was described in detail previously [18,19].

Before and after the completion of the training program, the chosen body mass and composition parameters were assessed via the bioimpedance method using electronic scale Tanita TBF 300 M (Arlington Heights, IL, USA), and biochemical analyses of blood samples were performed [20]. The following parameters were selected for this study: body mass index (BMI), fat mass (FM, kg), fat-free mass (FFM, kg), total body water (TBW, kg), total cholesterol (TC, mg/dL), triglycerides (TGL, mg/dL), high-density lipoprotein (HDL, mg/dL), low-density lipoprotein (LDL, mg/dL), and blood glucose level (BG, mg/dL).

The experiment was approved by the Ethics Committee of the Regional Medical Chamber in Szczecin (no. 09/KB/IV/2011 and 01/KB/VI/2017). Participants obtained an information sheet about the aim, procedures, benefits, and risks of the experiment, and a written consent form. Pseudonymization was applied as the method of data protection.

### 2.3. Total Genetic Score

First, the average values of the analyzed variables were compared by calculating their relative change by subtracting the variable’s value after training from the value of the variable before training. Second, the obtained results were used to create a polygenic profile for predicting post-training effects based on genotype data. According to Williams and Folland [21], each polymorphism site used to calculate the TGS was assigned a score based on the observed genotype (genotype score, GS). Typically, the polymorphisms identified were biallelic, providing 3 possible genotypes assigned GS values of 0, 1, or 2. The ‘optimal’ genotypes associated with beneficial post-training changes in selected variability (meaning that carriers of this genotype showed a relative change in the average values of the analyzed variables closest to the desired post-training effects) were scored 2, ‘intermediate’ genotypes were scored 1, and ‘less optimal’ genotypes (carriers of this genotype showed a change in the mean values of the analyzed variables that were farthest from the desired post-training effects) were scored 0. Due to the low abundance of one of the genotypes, homozygotes of one type were combined with heterozygotes for 6 polymorphisms. These patients only had two possible genotypes: a score of 2 or 0, indicating the least and most ‘optimal’ genotype. A decreasing BMI, FM, TC, TGL, LDL, and BG and increasing FFM, TBW, and LDL were considered favorable changes.

Afterward, the scores of each genotype were summed to generate the total score. The total score was converted to a scale of 0 to 100. The formula for calculating the TGS is as follows:

TGS = (100/(2 × number of analyzed variables)) × (GS_BMI_ + GS_FM_ + GS_FFM_ + GS_TBW_ + GS_TC_ + GS_TGL_ + GS_LDL_ + GS_HDL_ + GS_BG_)

Greater TGS values indicate a more favorable polygenic profile. Specifically, a TGS of 100 represents a ‘perfect’ profile when the genotype is the best for achieving all investigated post-training effects (all GS values calculated for this genotype are equal to 2), and a TGS of 0 represents the ‘worst’ possible profile in terms of achieving the expected effects (all GS values calculated for this genotype are equal to 0). The words ‘perfect’ and ‘worst’ are, of course, only to be interpreted within the context of this paper. All the other TGS values, ranging between 0 and 100, illustrate the intermediate value of a given genotype in the context of achieving the expected post-training effects.

### 2.4. Biofilter 

Biological information derivation and pairwise interaction modeling Biofilter 2.4 software [22] was used to derive biological information and construct pairwise interaction models. Initially, a list of SNPs was input into Biofilter software, which subsequently mapped these SNPs to corresponding genes. Next, genes harboring SNPs of interest were interconnected pairwise to explore common sources and groups defined within the Library of Knowledge Integration (LOKI) database. The LOKI database encompasses genes from multiple database repositories [22]. This integration facilitated the identification of common sources and groups among genes harboring relevant SNPs. Subsequently, the gene models were deconstructed into pairwise combinations of SNPs across genes, specifying the number of LOKI sources and the corresponding groups supporting these models. Each SNP within the interaction pairs was annotated with information from LOKI sources, and common entities were meticulously selected for group characterization.

### 2.5. Statistical Analysis of SNP Pairs and Interaction Testing

SNP pairs displaying potential interactions supported by biological knowledge were subjected to statistical interaction testing. A mixed-effects model in R (https://www.R-project.org/ (accessed on 19 January 2024) [23], specifically the ‘lme4’ package, version 1.1-31) was utilized to assess the impact on all obesity-related parameters. The significance of interactions was assessed through nested models, incorporating models with and without the interaction term and employing a likelihood ratio test. For models demonstrating a statistically significant interaction effect, predictor effect plots were generated using the ‘effects’ (version 4.2-2) library in R.

## 3. Results

### 3.1. Total Genetic Score

The complete list of the 30 SNPs localized in 18 genes associated with obesity-related parameters, the sources of all the information, the relative changes in the mean values of the analyzed variables, the GS for individual parameters, the GS sum, and the TGS values received for each genotype can be viewed in Table 1. The observed TGS values were in the range of 11–94. The TGS values distinguished five groups of genotypes:

1. TGS ≥ 80—includes four of the most preferable genotypes for training-induced body changes
2. TGS of 60–79—includes 21 ‘preferable’ genotypes for training-induced body changes
3. TGS of 40–59—includes 30 ‘intermediate’ genotypes for training-induced body changes
4. TGS of 20–39—includes 22 ‘unpreferable’ genotypes for training-induced body changes
5. TGS < 20—includes five of the most ‘non-preferable’ genotypes for training-induced body changes

The obtained data indicate that the highest TGS values (Group 1) reflecting the most ‘optimal’ genotypes in the context of obtaining all the desired post-training effects were obtained for the following genotypes: *ADRA2A* rs553668 AA (genotype frequency 3%; 94 TGS), *LEPR* rs1137101 AA (genotype frequency 29%; 94 TGS), *DRD2* rs1076560 AA (genotype frequency 2%; 83 TGS), and *LEP* rs2167270 GG (genotype frequency 40%; 83 TGS). The lowest TGS values (Group 5) reflecting the ‘worst’ possible profile in terms of achieving the expected effects were obtained for the following genotypes: *DRD2* rs1076560 CA (genotype frequency 28%; 11 TGS), *ADIPOQ* rs266729 GG (genotype frequency 6%; 17 TGS), *ADRB3* rs4994 TT (genotype frequency 86%; 17 TGS), *ADRA2A* rs553668 GG (genotype frequency 66%; 17 TGS), and *LEPR* rs1137101 AG (genotype frequency 49%; 17 TGS) (Table 1). The other genotypes presented intermediate TGS values.

**Table 1:** Polygenic profile of post-training changes in obesity-related parameters according to score.

Gene	SNP	Genotype	BMI		FM		FFM		TBW		TC		TGL		LDL		HDL		BG		Total GS	TGS
			∆_BMI_	GS_BMI_	∆_FM_	GS_FM_	∆_FFM_	GS_FFM_	∆_TBW_	GS_TBW_	∆_TC_	GS_TC_	∆_TGL_	GS_TGL_	∆_LDL_	GS_LDL_	∆_HDL_	GS_HDL_	∆_BG_	GS_BG_		
*ACE* [24]	rs1799752	II	−0.4	2	−0.9	0	0.4	0	0.3	0	−0.2	1	1.8	2	1	0	−1	2	−5	2	9	50
		ID	−0.1	0	−0.9	0	0.5	2	0.4	2	−1.7	2	2.8	1	2.4	1	−4.5	1	−1.6	1	10	56
		DD	−0.2	1	−1.2	2	0.5	2	0.4	2	1.1	0	6.5	0	4.7	2	−4.9	0	−0.5	0	9	50
*ADIPOQ* [18]	rs266729	CC	−0.23	2	−0.91	2	0.4	2	0.1	1	0.16	1	6.65	1	4.93	1	−4.3	0	−2.74	2	12	67
		GC	−0.2	1	−0.83	1	0.25	0	0.24	2	−5.65	2	−4.78	2	−2.23	2	−2.95	2	−1.73	0	12	67
		GG	−0.05	0	−0.24	0	0.32	1	−0.17	0	3.24	0	9.84	0	36.1	0	−4.07	1	−2.08	1	3	17
	rs1501299	GG	−0.14	0	−0.46	0	0.18	0	0.04	1	1.26	0	6.56	1	12.61	0	−3.85	2	−2.97	2	6	33
		TG	−0.26	2	−1.04	1	0.42	1	0.39	2	−4.15	2	−1.58	2	2.11	1	−5.36	0	−2.08	1	12	67
		TT	−0.2	1	−1.32	2	0.69	2	−0.78	0	−4	1	7.16	0	−5.14	2	−3.86	1	−0.1	0	9	50
*ADRB2* [25]	rs1042713	GG	−0.12	0	−0.92	0	0.39	0	0.46	2	−0.59	1	4.79	1	2.67	1	−3.56	2	−2.96	1	8	44
		AG	−0.31	1	−1.61	1	0.43	1	−0.25	0	−2.12	2	0.51	2	2.44	2	−4.75	0	−3.36	2	11	61
		AA	−0.32	2	−2.07	2	0.6	2	0.4	1	0.05	0	11.09	0	40.65	0	−3.8	1	−0.73	0	8	44
	rs1042714	GG	−0.14	0	−1.07	0	0.45	2	0.32	2	−2.71	1	−2.21	2	2.04	2	−3.57	1	−2.47	2	12	67
		CG	−0.23	1	−1.28	1	0.42	0	−0.05	0	2.34	0	8.17	0	5.29	1	−4.99	0	−2.41	1	4	22
		CC	−0.3	2	−1.69	2	0.44	1	0.3	1	−4.61	2	2.45	1	15.13	0	−3.28	2	−2.35	0	11	61
*ADRB3* [25]	rs4994	TT	0.5	0	−1.38	2	−0.47	0	0.15	0	−0.33	0	4.49	0	9.56	0	−4.81	0	−2.36	1	3	17
		CT	−0.29	2	−1.36	1	0.25	1	0.18	1	−3.54	1	0.67	1	−2.66	2	−0.62	1	−2.04	0	10	56
		CC	0.03	1	−0.84	0	0.57	2	0.37	2	−3.67	2	0.66	2	6.5	1	0.73	2	−7.33	2	14	78
*ADRA2A* [25]	rs553668	GG	−0.24	1	−1.21	0	0.36	0	0.22	1	−1.58	1	4.56	0	9.37	0	−4.82	0	−2.33	0	3	17
		AG	−0.18	0	−1.86	1	0.65	1	−0.08	0	0.95	0	3.3	1	2.75	1	−1.78	2	−2.41	1	7	39
		AA	−0.45	2	−1.9	2	0.9	2	0.75	2	−12	2	−24.5	2	−3.9	2	−1.9	1	−7	2	17	94
*AMPD1* [26]	rs17602729	CC	−0.2	0	−0.9	0	0.4	0	0.3	0	−4	2	2	0	−1	0	−3	2	−3	2	6	33
		TT + CT	−0.3	2	−1	2	0.5	2	0.4	2	1	0	1	2	−5	2	−5	0	−2	0	12	67
*DRD2* [27]	rs1076560	AA	−0.57	2	−1.9	2	0.85	2	0.79	2	−5.5	2	−8.17	2	2.48	1	−6.21	0	−8.84	2	15	83
		CA	−0.18	0	−0.89	0	0.28	0	0.05	0	−1.08	0	10.3	0	2.81	0	−5.89	1	−3.79	1	2	11
		CC	−0.23	1	−0.91	1	0.47	1	0.44	1	−1.77	1	0.79	1	1.3	2	−3.14	2	−1.88	0	10	56
	rs12364283	AA	−0.22	1	−0.98	2	0.46	2	0.36	2	−0.75	0	3.92	1	2.49	0	−3.96	2	−2.73	1	11	61
		GA	−0.29	2	−0.76	1	0.23	1	0.18	1	−8.25	1	−2.94	2	−2.86	1	−4.6	1	−1.88	0	10	56
		GG	−0.1	0	0.25	0	−0.6	0	−0.45	0	−19	2	7	0	−12.2	2	−7.7	0	−7	2	6	33
	rs1799732	C–	−0.26	2	−0.94	0	0.26	0	0.27	0	−4.79	2	−5.37	2	−0.8	2	−2.83	2	−4.84	2	12	67
		CC	−0.22	0	−0.95	2	0.49	2	0.36	2	−0.78	0	5.88	0	2.57	0	−4.44	0	−2.06	0	6	33
	rs1800497	CC	−0.21	0	−0.83	0	0.46	1	0.45	1	−1.74	1	0.99	1	1.4	2	−3.26	2	−1.71	0	8	44
		CT	−0.24	1	−1.1	1	0.36	0	0.12	0	−1.1	0	8.53	0	2.43	1	−5.16	1	−3.95	1	5	28
		TT	−0.54	2	−1.4	2	0.58	2	0.62	2	−7.6	2	−9	2	2.6	0	−8.24	0	−9	2	14	78
	rs1800498	CC	−0.21	0	−0.88	1	0.75	2	0.6	2	0.38	0	7.58	0	1.04	2	−2.05	2	−5.35	2	11	61
		TC	−0.22	1	−1.03	2	0.43	1	0.2	0	−3.75	2	−0.55	2	1.79	1	−5.35	0	−2.99	1	10	56
		TT	−0.24	2	−0.49	0	0.21	0	0.38	1	−0.02	1	6.19	1	2.27	0	−3.46	1	−0.59	0	6	33
*FABP2* [28]	rs1799883	GG	−0.2	0	−1.1	2	0.5	2	0.5	2	−4	2	1.5	2	0.3	2	−4.5	0	−3.2	2	14	78
		GA+AA	−0.3	2	−0.9	0	0.3	1	0.2	0	1	0	5.1	0	4.1	0	−3.3	2	−2.1	0	5	28
*FTO* [20]	rs9939609	TT	−0.3	2	−1	2	0.45	2	0.1	0	−0.3	0	3.9	0	4	0	−3.8	0	−2.6	2	8	44
		AA+AT	−0.2	0	−0.9	0	0.4	0	0.5	2	−2.3	2	2.8	2	1.1	2	−3.6	2	−2.2	0	10	56
*IL1A* [29]	rs1800587	TT	−0.3	2	−1.1	2	0.6	2	0.5	2	0	0	4	1	3	1	−3	2	−3	1	13	72
		CT	−0.2	0	−0.7	0	0.4	1	0.2	1	−2	1	2	2	2	2	−4	1	−2	0	8	44
		CC	−0.25	1	−1	1	0.3	0	−0.4	0	−10	2	11	0	7	0	−7	0	−6	2	6	33
*IL6* [29]	rs1800795	GG	−0.3	2	−0.8	0	0.3	0	0.3	0	−2	2	−1.3	2	2.3	0	−4	1	−2.2	0	7	39
		CG	−0.2	1	−1.1	2	0.5	1	0.3	0	−1	0	6.8	0	2.3	0	−5	0	−2.9	2	6	33
		CC	−0.1	0	−0.9	1	0.8	2	0.5	2	−1	0	3.4	1	−0.1	2	−1.6	2	−2.7	1	11	61
	rs1800796	GG	−0.3	2	−1	2	0.45	2	0.4	0	−2	2	3.4	2	0.8	2	−3.8	2	−2.4	0	14	78
		CC+CG	−0.2	0	−0.8	0	0.4	0	0.45	2	2	0	3.8	0	7.2	0	−5.2	0	−3.7	2	4	22
	rs1800797	GG	−0.2	1	−0.7	0	0.7	1	0.3	0	−1	1	−0.4	2	3.9	0	−4.5	1	−2.5	1	7	39
		AG	−0.3	2	−1.1	2	0.4	0	0.3	0	−3	2	6.2	0	0.6	2	−4.8	0	−3.2	2	10	56
		AA	0	0	−0.8	1	0.8	2	0.6	2	0	0	3.5	1	1	1	−1.4	2	−1.7	0	9	50
*IL15* [19]	rs1589241	AA	−0.2	1	−0.9	0	0.4	1	0.4	1	1	0	0.4	2	4.5	0	−3.4	2	−	−	7	44
		AT	−0.25	2	−1	1	0.3	0	0.1	0	−3	1	0.5	1	−0.5	2	−4.6	1	−	−	8	50
		TT	−0.1	0	−1.2	2	1.1	2	0.8	2	−4	2	0.9	0	0.7	1	−6.3	0	−	−	9	56
	rs1057972	TT	−0.2	0	−0.6	0	0.1	0	0.3	0	2	0	0.1	2	6.3	0	−3.3	2	−	−	4	25
		AT	−0.25	1	−0.9	1	0.6	2	0.3	0	−3	2	0.4	1	0.1	2	−4.9	0	−	−	9	56
		AA	−0.3	2	−1.4	2	0.6	2	0.4	2	0	1	1	0	1.2	1	−3.5	1	−	−	11	69
*LEP* [30]	rs2167270	GG	−0.3	2	−1.2	2	0.45	2	0.4	1	−3	2	0.3	2	−0.1	2	−2.8	1	−1.1	1	15	83
		AG	−0.2	0	−0.8	0	0.4	0	0.3	0	−3	2	4.9	1	1.9	1	−5.5	0	−5.3	2	6	33
		AA	−0.2	0	−1.1	1	0.4	0	0.6	2	4	0	6.2	0	5.8	0	−2.6	2	0.6	0	5	28
*LEPR* [30]	rs1137101	AA	−0.25	1	−1.2	2	0.7	2	0.6	2	−6	2	1.5	2	−3.1	2	−3	2	−3	2	17	94
		AG	−0.2	0	−0.8	0	0.3	0	0.4	1	1	0	4.7	0	4.5	0	−3.8	1	−2.6	1	3	17
		GG	−0.3	2	−1	1	0.3	0	0	0	−2	1	2.4	1	2.3	1	−5.4	0	−2.1	0	6	33
*MC4R* [31]	rs17782313	TT	−0.2	0	−0.95	0	0.4	0	0.3	2	−2	2	3.3	2	1.2	2	−3.8	2	−2.01	0	10	56
		CC+CT	−0.3	2	−1.25	2	0.5	2	−0.1	0	1	0	8.6	0	15.7	0	−5.1	0	−3	2	8	44
*PPARA* [32]	rs4253778	GG	−0.23	1	−0.88	0	0.41	0	0.24	0	−1.95	2	3.37	1	0.37	2	−2.88	2	−2.39	1	9	50
		GC	−0.24	2	−1.08	1	0.46	1	0.48	1	−1.77	1	5.4	0	3.06	1	−5.89	1	−4.28	2	10	56
		CC	−0.22	0	−1.22	2	0.55	2	0.7	2	7.67	0	−12	2	16.31	0	−6.52	0	7.67	0	8	44
	rs18000206	CC	−0.21	0	−0.88	0	0.43	0	0.34	2	−1.63	2	4.26	0	0.95	2	−3.34	2	−3.09	2	10	56
		CG	−0.45	2	−1.65	2	0.45	2	0.32	0	−0.71	0	−5.43	2	11	0	−10.79	0	2.35	0	8	44
*PPARD* [33]	rs2267668	AA	−0.25	2	−1.39	1	0.33	0	0.02	0	0.88	0	7.74	0	3.54	1	−4.05	1	−2.54	1	6	33
		AG	0.2	1	−1.66	2	0.56	1	0.49	1	−5.86	1	−12.9	2	29.24	0	−3.22	2	−0.04	0	10	56
		GG	0.27	0	0.53	0	1.93	2	1.47	2	−19.66	2	6.17	1	−11.42	2	−9.65	0	−10.33	2	11	61
	rs2016520	TT	−0.25	2	−1.4	1	0.34	0	0.01	0	0.77	0	8.11	0	3.75	1	−4.43	1	−2.68	1	6	33
		TC	0.2	1	−1.65	2	0.58	1	0.48	1	−4.6	1	−12.69	2	67.08	0	−1.8	2	0.28	0	10	56
		CC	0.22	0	0.61	0	1.54	2	1.19	2	−18.72	2	4.72	1	−10.22	2	−8.85	0	−8.86	2	11	61
	rs1053049	TT	−0.22	2	−12.35	2	0.38	1	−0.05	0	−0.12	0	8.66	0	3.19	1	−4.6	1	−2.77	1	8	44
		TC	0.01	1	−1.71	1	−1.96	0	0.57	1	−1.68	1	−7.11	1	21.09	0	−2.22	2	−1.02	0	7	39
		CC	0.16	0	1.3	0	1.56	2	1.22	2	−19.4	2	−8	2	−6.52	2	−9.24	0	−5.2	2	12	67
*TNF-α* [34]	rs1800629	GG	−0.21	0	−0.94	0	0.51	2	0.31	0	−1.62	2	1.26	2	3.82	0	−5.63	0	−1.98	0	6	33
		AA+AG	−0.27	2	−0.96	2	0.26	0	0.4	2	−1.4	0	8.11	0	−2.52	2	0.43	2	−4	2	12	67

∆—change in variable (before and after the completion of the training program); SNP—single nucleotide polymorphism; GS—genotype score; TGS—total genotype score; BMI—body mass index; FM—fat mass; FFM—fat-free mass; TBW—total body water; TC—total cholesterol; TGL—triglycerides; LDL—low-density lipoprotein; HDL—high-density lipoprotein.

### 3.2. Biofilter

Using Biofilter with the LOKI database, five noteworthy pairwise interactions were identified, each revealing potential associations between specific genes and their corresponding SNPs (Table 2).

**Figure 1:**
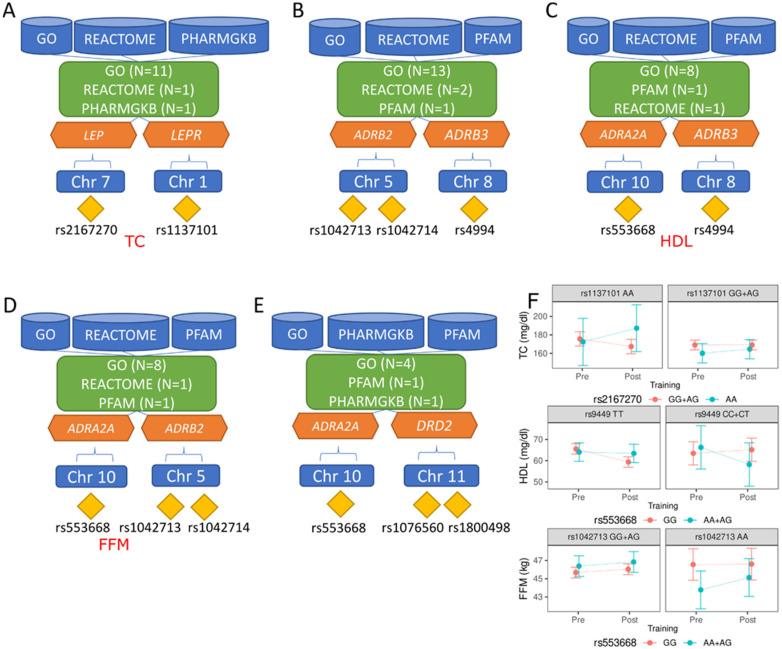
Detailed information about the sources and groups for each interaction SNP pair. (**A**) *LEP* × *LEPR*: GO—angiogenesis, positive regulation of protein phosphorylation, protein binding, energy reserve metabolic process, negative regulation of autophagy, sexual reproduction, T cell differentiation, leptin-mediated signaling pathway, regulation of bone remodeling, bone growth, positive regulation of cold-induced thermogenesis; PHARMGKB—Antipsychotics Pathway (Metabolic Side Effects), Pharmacodynamics; REACTOME—R-HSA-2586552; (**B**) *ARDB2* × *ADRB3*: GO—norepinephrine–epinephrine-mediated vasodilation involved in regulation of systemic arterial blood pressure, desensitization of G-protein-coupled receptor signaling pathway by arrestin, protein binding, plasma membrane, receptor-mediated endocytosis, adenylate-cyclase-modulating G-protein-coupled receptor signaling pathway, activation of adenylate cyclase activity, protein homodimerization activity, receptor complex, positive regulation of MAPK cascade, norepinephrine binding, adenylate-cyclase-activating adrenergic receptor signaling pathway, positive regulation of cold-induced thermogenesis; PFAM—7 transmembrane receptor (rhodopsin family); REACTOME—R-HSA-390696, R-HSA-418555; (**C**) *ADRA2A* × *ADRB3* GO—protein binding, plasma membrane, protein homodimerization activity, receptor complex, positive regulation of MAPK cascade, epinephrine binding, norepinephrine binding, adenylate-cyclase-activating adrenergic receptor signaling pathway; PFAM—7 transmembrane receptor (rhodopsin family); REACTOME—R-HSA-390696; (**D**) *ADRA2A* × *ADRB2*: GO—protein binding, plasma membrane, protein homodimerization activity, receptor complex, positive regulation of MAPK cascade, norepinephrine binding, adrenergic receptor signaling pathway, adenylate-cyclase-activating adrenergic receptor signaling pathway; PFAM—7 transmembrane receptor (rhodopsin family); REACTOME—R-HSA-390696; (**E**) *ADRA2A* × *DRD2*: GO—protein binding, plasma membrane, heterotrimeric G-protein binding, adenylate-cyclase-activating adrenergic receptor signaling pathway; PFAM—7 transmembrane receptor (rhodopsin family); PHARMGKB—Methylphenidate Pathway, Pharmacodynamics; (**F**) predictor effect plots, providing insights into the relationships between changes in predictor variables and corresponding alterations in the predicted response variable; GO (Gene Ontology)—GO is a comprehensive bioinformatics resource that provides structured and standardized terms to describe the functions of genes and proteins in any organism. It categorizes gene functions into three main ontologies: Molecular Function (the molecular activities of gene products), Biological Process (the larger biological goals accomplished by gene products), and Cellular Component (the locations in the cell where gene products are active); PFAM—widely used database that classifies proteins into families based on the presence of specific conserved protein domains or functional units. It provides information about the structure and function of these protein domains; REACTOME—curated and peer-reviewed pathway database that provides insights into biological pathways, reactions, and biomolecule interactions. It covers many biological processes, including signaling pathways, metabolic pathways, and immune system responses.

Three sources supported all interactions; the groups ranged from 6 to 16. Detailed information about the sources and groups for each interaction SNP pair is shown in Figure 1A–E. Based on this, comprehensive analyses were conducted to explore the implications of pairwise interactions suggested by Biofilter software, employing mixed-effect models for each obesity-related parameter. The investigation identified three specific interactions demonstrating significant associations with key parameters crucial for metabolic health—TC, HDL, and FFM (see Figure 1F). Figure 1F shows predictor effect plots, providing insights into the relationships between changes in predictor variables and corresponding alterations in the predicted response variable. For the TC (the upper panel in Figure 1F), compound homozygotes with *LEP* rs2167270 AA and *LEPR* rs1137101 AA showed a more significant increase in TC than those with other genotypes. For HDL (the middle panel of Figure 1F), compound genotypes, such as *ADRA2A* rs553668 AA+AG and *ADRB3* rs9449 CC+CT, displayed a more pronounced decrease in HDL cholesterol during intervention than did the other genotypes. For FFM (the bottom panel in Figure 1F), we explored the interaction between *ADRA2A* GG and AA+AG based on baseline differences in AA homozygotes in *ADRB2*. The interaction of FFM, while detected, appears to be of lesser importance in the context of training response, as it depends on baseline differences in AA homozygotes in *ADRB2* between the *ADRA2A* GG and AA+AG genotypes.

## 4. Discussion

According to studies performed in twins, families, and adoptees, the heritability of body mass status ranges from 40% to 50%. However, this value is lower among normal-weight individuals (approximately 30%) and higher in obese people (60–80%) [35]. Genetic epidemiological studies have shown that numerous genetic loci identified by genome-wide association studies (GWASs) cumulatively increase the risk of obesity [17] and may influence physical activity and sedentary behavior in daily life [36]. Although genetically predisposed individuals are more prone to developing obesity, it has been shown that the level of physical activity can modify the genetic predisposition to common obesity. Li et al. [37] indicated that a physically active lifestyle is associated with a 40% reduction in genetic susceptibility to obesity. The authors emphasized the importance of promoting exercise, particularly in genetically predisposed individuals, as a significant approach to controlling the growing obesity epidemic [37]. However, we still do not know whether or to what extent habitual physical activity may weaken this genetic susceptibility [37]. Therefore, my research on gene–physical-activity interactions in obesity for 10 years has resulted in more than 15 published articles [15,18,19,20,24,25,26,27,28,29,30,31,32,33,34]. Because the individual studies involved only single or small numbers of polymorphisms, which did not allow for their simultaneous analysis to elucidate the complex associations between the genetic determinants of obesity and physical activity, previously obtained results were used to describe their impact on post-training response comprehensively.

The most important achievement of this work was identifying specific genotypes associated with favorable or undesirable training-induced changes in body mass, body composition, and biochemical parameters (‘preferable’, ‘intermediate’, and ‘unpreferable’, respectively). The data obtained showed that the most significant impact on the effectiveness of a 12-week training program involved genes encoding leptin and a leptin receptor (*LEP* and *LEPR*), adrenergic receptors (*ADRA2A* and *ADRB3*), a dopamine receptor D2 (*DRD2*), and an adiponectin receptor (*ADIPOQ*). None of the genotypes achieved the TGS value of either 0 or 100, reflecting the ‘worst’ or the ‘perfect’ profile. The best result was 94 TGS for the *ADRA2A* rs553668 AA and *LEPR* rs1137101 AA genotypes, which indicated that carriers of these genotypes exhibited a beneficial change in the average values of all the analyzed variables (eight parameters were scored 2, and one was scored 1). In addition, two genotypes, *DRD2* rs1076560 AA and *LEP* rs2167270 GG, had a TGS of 83 and were classified as the most ‘preferable’ genotypes for training-induced body changes. The worst result was 11 TGS for *DRD2* rs1076560 CA, and the carriers of this genotype exhibited an unbeneficial change in the mean values of almost all the chosen variables (seven parameters were scored 0, and two were scored 1). The group of individuals with the most ‘unpreferable’ genotypes for training-induced body changes also included genotypes such as *ADIPOQ* rs266729 GG, *ADRB3* rs4994 TT, *ADRA2A* rs553668 GG, and *LEPR* rs1137101 AG, for which the TGS value was 17. These results confirm the role of the various genes in the development of obesity described by several authors [2,13,32]. However, a polygenic profile aimed at finding all the ‘preferable’ and ‘unpreferable’ genotypes for training-induced body changes was created for the first time. Thus, this study cannot be directly compared to previous studies.

In the 2000s, GWASs allowed the analysis of polymorphic sites in the whole genome to link common and low-frequency genomic variants to phenotypes such as obesity. The first defined obesity susceptibility gene with a more significant influence on body mass to date was the fat mass and obesity-associated (*FTO*) gene. The common *FTO* polymorphism with a T-to-A change (rs9939609) is strongly associated with an increased risk of obesity development in various populations. Each A allele, a risk allele, is associated with a 1–1.5 kg increase in body weight [38]. My interventional study confirmed that participants with the AA and AT genotypes had increased BMIs during the entire study period; however, the *FTO* gene–physical-activity interaction was not demonstrated [20]. This analysis confirmed that no *FTO* genotype was associated with better or worse training-induced body changes. Thus, not all obesity-related polymorphisms are crucial for assessing the effectiveness of weight loss programs. Some studies have been unable to demonstrate this interaction [39,40]; however, others have shown that the effect size of *FTO* variants is up to 80% lower in physically active individuals than in inactive individuals [41,42]. Other SNPs in the *LEP*, *LEPR*, *ADIPOQ*, *ADRA2A*, and *ADRB3* genes, which are described as key for energy intake and fat metabolism [2], are also important in the body’s adaptive response to training. Adipose tissue plays significant roles in body weight regulation and energy homeostasis, including the production and secretion of numerous cytokines, chemokines, and hormone-like factors known as adipokines [43]. Leptin and adiponectin are critical in food intake, metabolism, and immunity. Leptin, which acts as an afferent signal in a negative feedback loop by binding to the leptin receptor, plays important roles in regulating body weight by suppressing appetite and stimulating energy expenditure [44,45]. Adiponectin is an essential anti-inflammatory and insulin-sensitizing hormone that promotes lipid oxidation in tissues such as skeletal muscle and liver [46]. The brain receives signals from adipose tissue, which activates neural circuits controlling energy expenditure and increases sympathetic nerve activity. Adrenergic receptors are part of the sympathetic nervous system and exert their actions by coupling with catecholamines, which are important regulators of lipolysis and energy expenditure [47]. Considering the multifactorial role of these gene products in regulating energy metabolism, it was expected that they would also contribute to the post-training response, which was confirmed by the analysis. Surprisingly, the genotypes of the rs1076560 polymorphism in the *DRD2* gene encoding the dopamine receptor D2 were the genotypes with the highest post-training effect. Although it is associated with essential central nervous system functions, such as cognitive abilities, its impact on exercise-induced changes has rarely been analyzed, making comparison difficult. This analysis highlights that the genes related to the dopaminergic system may play a significant role in the effectiveness of training programs, so continuing research is necessary.

The second part of this analysis, conducted using Biofilter software, confirmed the biological significance of the SNPs. These SNPs are most important, based on the TGS, for the changes in body mass, composition, and biochemical parameters induced by training. Five noteworthy pairwise interactions (*LEP* × *LEPR*, *ADRB2* × *ADRB3*, *ADRA2A* × *ADRB3*, *ADRA2A* × *ADRB2*, and *ADRA2A* × *DRD2*) were identified. The core principle guiding the analysis conducted with Biofilter software is that any grouping of genes or proteins, whether a pathway, ontological category, protein family, experimental interaction, or any other classification, implies a potential relationship among the individual elements within that group. When the same two genes repeatedly appear together in various groupings, this signifies a substantial biological relationship. Furthermore, if these genes are present in multiple groups sourced from diverse, independent origins, their probability of being biologically related significantly increases. Biofiltering taps into an extensive repository of such groupings, facilitating the examination of all these associations. The tool discerns pairs of genes or SNPs that co-occur across numerous groupings spanning various original data sources. Consequently, these gene pairs can undergo significance testing within a research dataset, eliminating the need for exhaustive pairwise analyses that would pose computational challenges and the burden of multiple testing. Based on these findings, comprehensive analyses were conducted to explore the implications of pairwise associations suggested by Biofilter software, employing mixed-effect models for each obesity-related parameter. Three specific interactions demonstrating significant associations with key parameters crucial for metabolic health—TC, HDL cholesterol, and FFM—were shown. Carriers of the *LEP* rs2167270 AA and *LEPR* rs1137101 AA exhibited greater increases in TC levels during the intervention, suggesting that the average values of these parameters did not change with age. Compound genotypes such as *ADRA2A* rs553668 AA+AG and *ADRB3* rs9449 CC+CT showed a more substantial training-induced decrease in HDL levels and may be classified as unpreferable genotypes. The third interaction, detected for FFM, appears to be of minor importance in training response, as it depends on baseline differences in AA homozygotes in *ADRB2* between the *ADRA2A* GG and AA+AG genotypes. These findings highlight the nuanced relationships between genetic variations and metabolic health parameters, providing insights into potential factors that influence individual responses to training interventions.

GWASs have recognized numerous genetic loci associated with lipid traits. However, these loci explain only 25–30% of the heritability observed at the blood lipid level [48]. Interactions between genes may explain a part of this missing heritability [49]. Previously, Holzinger et al. [50] performed a gene-centric interaction study for four different lipid traits, LDL, HDL, TC, and BG, using a main-effect filter and biofilters. More models passed the selected replication threshold for the main-effect filter analyses. However, for the biofilter analyses, the results were replicated only for the BG trait, with two models passing the significance threshold in a single cohort (*SIK3* rs11216162 × *APOA4* rs1263173 and *SIK3* rs625145 × *APOA4* rs1263173). The authors suggested that biofilter analysis, which creates gene–gene models based on current biological knowledge, allows for more precise interpretations, as the models make biological sense. However, this approach inhibits the discovery of interactions in regions with limited biological knowledge. Using the genetic data from five cohorts of 24,837 individuals, De et al. [51] combined the quantitative multifactor dimensionality reduction algorithm with two SNP filtering methods to search for interactions between SNPs linked to lipid traits. When SNPs were filtered using Biofilter, two models associated with HDL cholesterol, three associated with LDL cholesterol, one associated with TC, and eight associated with BG were revealed. However, none of these interactions were consistent with the results obtained in this study. These differences may be explained by the fact that previous studies did not address changes in post-training parameters. To the best of my knowledge, this is the first study to analyze the interactions between SNPs in the context of post-training changes in selected parameters, which makes it difficult to compare the results.

The strong point of the study was the comprehensive analysis of numerous genetic data obtained from the experiment consisting of regulation of both food intake and physical activity of a homogeneous Caucasian population, whose body mass and composition, as well as physiological and biochemistry parameters, were analyzed before and after the completion of the 12-week training program. In addition, the use of creative approaches such as the construction of a polygenic profile and Biofilter software has provided novel insights into potential factors influencing individual responses to training interventions. It needs to be highlighted that common obesity is a multifactorial disease, most likely resulting from a complicated interaction of genetic, epigenetic, and environmental components [52,53]. Among the many factors influencing response to exercise training are age, gender, diseases, volume, intensity and frequency of exercise, diet, and many others [16]. Therefore, a potential limitation of the study is the small size of the participant group, which may not show statistical power sufficient to yield meaningful analysis and interpretation. Another factor that should be considered as a weakness is population-specific characteristics such as one gender, similar age, relatively high physical activity levels as well as a relatively low weight. Previously, studies investigating gene-by-sex interactions for obesity have shown an interplay between sex and obesity-related traits, and specific polymorphisms can be associated with obesity in one sex [54]. Unfortunately, the experiment only included young Caucasian women, and thus there was no possibility of comparing the results between genders, ethnicities, and age groups. Additionally, Aurich et al. have shown that intervention studies documenting changes in a systemic epigenetic biomarker for obesity susceptibility during weight loss programs make a significant contribution to a better understanding of epigenetic reprogramming in obesity [52]. Epigenetic modifications include DNA methylation, histone modifications, and non-coding RNAs (microRNAs, miRs) which mediate between environmental and genetic factors. These alterations may be causal for the development of obesity by inducing improper expression or silencing of the obesity-associated genes and regulatory sequences, leading to metabolic balance disorders. Epigenetic changes can also arise as a consequence of obesity and predispose for obesity-associated comorbidities such as cancer [52,55,56,57]. This study did not examine lifestyle effects on epigenetic remodeling, which is another weak point of the study.

## 5. Conclusions

In this study, two novel approaches, total genetic score and Biofilter software, were used to combine genotypic and phenotypic information for nine obesity-related traits measured before and after the initiation of a 12-week aerobic training program. The first important finding was the indication of ‘preferable’, ‘intermediate’, and ‘unpreferable’ genotypes for training-induced changes in selected body mass, composition, and biochemical parameters. The genes most important for post-workout changes were *LEP*, *LEPR*, *ADIPOQ*, *ADRA2A*, *ADRB3*, and *DRD2*. The second finding involved the identification of five noteworthy pairwise interactions (*LEP* × *LEPR*, *ADRB2* × *ADRB3*, *ADRA2A* × *ADRB3*, *ADRA2A* × *ADRB2*, and *ADRA2A* × *DRD2*) and three specific interactions demonstrating significant associations with key parameters crucial for metabolic health—TC, HDL, and FFM. Understanding the genetic architecture and its interactions with lifestyle factors such as physical activity level enables us to clarify individuals’ physical activity criteria. In the future, training programs designed according to a given person’s genetic profile will be effective and safe intervention strategies for preventing obesity and improving health.

## Figures and Tables

**Table 2:** SNP interactions with scores supported by Biofilter modeling.

Gene × Gene	SNP 1 × SNP 2	Score	
		Source	Group
*LEP* × *LEPR*	rs2167270 × rs1137101	3	13
*ADRB2* × *ADRB3*	rs1042713 × rs4994	3	16
	rs1042714 × rs4994	3	16
*ADRA2A* × *ADRB3*	rs553668 × rs4994	3	10
*ADRA2A* × *ADRB2*	rs553668 × rs1042713	3	10
	rs553668 × rs1042714	3	10
*ADRA2A* × *DRD2*	rs553668 × rs1076560	3	6
	rs553668 × rs1800498	3	6

The score is a combination of two tallies: the number of original data sources which contained the pair and the number of different groups among those sources. For example, a score of “3–13” indicates that the model appears in thirteen different groups, and those groups originated with three different sources.

## Data Availability

The data presented in this study are available on request from the author. The data are not publicly available due to privacy/ethical restrictions.
